# Protective Effects of *Lactobacillus plantarum* 16 and Paenibacillus polymyxa 10 Against *Clostridium perfringens* Infection in Broilers

**DOI:** 10.3389/fimmu.2020.628374

**Published:** 2021-02-18

**Authors:** Li Gong, Baikui Wang, Yuanhao Zhou, Li Tang, Zihan Zeng, Huihua Zhang, Weifen Li

**Affiliations:** ^1^ Key Laboratory of Molecular Animal Nutrition of the Ministry of Education, College of Animal Sciences, Institute of Feed Science, Zhejiang University, Hangzhou, China; ^2^ School of Life Science and Engineering, Foshan University, Foshan, China

**Keywords:** *Clostridium perfringens*, *Lactobacillus plantarum* 16, *Paenibacillus polymyx*a 10, broilers, apoptosis, immunity, microbiota, metabolic pathways

## Abstract

This study aimed to investigate the protective effects of *Lactobacillus plantarum* 16 (Lac16) and *Paenibacillus polymyx*a 10 (BSC10) against *Clostridium perfringens* (Cp) infection in broilers. A total of 720 one-day-old chicks were randomly divided into four groups. The control and Cp group were only fed a basal diet, while the two treatment groups received basal diets supplemented with Lac16 (1 × 10^8^ cfu·kg^−1^) and BSC10 (1 × 10^8^ cfu·kg^−1^) for 21 days, respectively. On day 1 and days 14 to 20, birds except those in the control group were challenged with 1 × 10^8^ cfu *C. perfringens* type A strain once a day. The results showed that both Lac16 and BSC10 could ameliorate intestinal structure damage caused by *C. perfringens* infection. *C. perfringens* infection induced apoptosis by increasing the expression of Bax and *p53* and decreasing Bcl-2 expression and inflammation evidence by higher levels of *IFN-γ, IL-6, IL-1β*, *iNOS*, and *IL-10* in the ileum mucosa, and NO production in jejunal mucosa, which was reversed by Lac16 and BSC10 treatment except for *IL-1β* (*P <* 0.05). Besides, the two probiotics restored the intestinal microbiota imbalance induced by *C. perfringens* infection, characterized by the reduced *Firmicutes* and *Proteobacteria* and the increased *Bacteroidetes* at the phyla level and decreased *Bacteroides fragilis* and *Gallibacterium anatis* at the genus level. The two probiotics also reversed metabolic pathways of the microbiota in *C. perfringens*-infected broilers, including B-vitamin biosynthesis, peptidoglycan biosynthesis, and pyruvate fermentation to acetate and lactate II pathway. In conclusion, Lac16 and BSC10 can effectively protect broilers against *C. perfringens* infection through improved composition and metabolic pathways of the intestinal microbiota, intestinal structure, inflammation, and anti-apoptosis.

## Introduction

Necrotic enteritis (NE) caused by *Clostridium perfringens* (Cp) is a severe gastrointestinal disease responsible for the annual loss of at least $ 6 billion in the poultry industry worldwide (1, 2). The disease usually occurs in two forms, including clinical and subclinical forms. The clinical form is characterized by a sudden rise in flock mortality with no premonitory signs, while the subclinical form is associated with intestinal damage, impaired absorption of nutrients, and poor overall performance in poultry (3). Moreover, as a common foodborne pathogen that affects humans, *C. perfringens* poses a serious threat to human health *via* contaminated poultry (4). Antibiotics have been used as the most effective measure to control *C. perfringens* infection in livestock (5). However, due to the emergence of antibiotic-resistant bacteria and the effect of antibiotics on the microbiome (6), there is an urgent need to find suitable alternatives to reduce the incidence of necrotic enteritis and maintain animal and human health in the post-antibiotics era.

Probiotics are live microorganisms, which when administered in adequate amounts exert their benefits on the host (7). The use of probiotics is a promising measure for the prevention of intestinal diseases, such as colitis (8), inflammatory bowel disease (9), and diarrhea (10). Besides, probiotics can protect the host against pathogen invasion by manipulating the complex gut ecosystems through competitive exclusion, production of antimicrobial compounds, stimulation of the host immune system development, *etc* (11). Fukata et al. (12) revealed that *Lactobacillus acidophilus* or *Streptococcus faecalis* could reduce *C. perfringens* infection in young chickens. Numerous other studies have also shown that some microorganisms, including *Bacillus* (13), *Lactobacilli* (14), *Enterococci* (15), and *yeast* (16), could alleviate the severity and damage of NE in chicken *via* different mechanisms. *Lactobacillus johnsonii* BS15 was reported to prevent NE by ameliorating lipid metabolism and intestinal microflora (14), while *Lactobacillus fermentum* 1.2029 regulated the expression levels of cytokines and TLR in chicken (17). Our previous studies also found that *Lactobacillus plantarum* Lac16 and *Paenibacillus polymyxa* BSC10 could inhibit the growth and virulence-associated gene expressions of *C. perfringens in vitro* (unpublished data) and regulate the mRNA expression of cytokines and TLR in chicken macrophage (HD11) cell line infected with *C. perfringens* (unpublished data). Therefore, the present study aimed to investigate the protective effects of Lac16 and BSC10 against *C. perfringens* in broilers.

## Materials and Methods

### Bacterial Strain Preparation

The *Lactobacillus plantarum* 16 (Lac16) (CCTCC, NO. M2016259) was isolated by our laboratory and preserved in China Center for Type Culture Collection. *Paenibacillus polymyxa 10* (BSC10) (CGMCC 1.10711) was obtained from the China General Microbiological Culture Collection Center. *Clostridium perfringens* (ATCC13124) was purchased from Guangdong Microbial Culture Collection Center.

Probiotics Lac16 and BSC10 were cultured in MRS or Luria–Bertani (LB) broth overnight at 37°C in an anaerobic system or a shaking incubator, respectively. *Clostridium perfringens* was cultured in Reinforced Clostridium Medium for 24 h under an anaerobic environment. The bacteria were harvested after centrifugation at 4,000 × g for 15 min at 4°C, respectively. After three-time washing with sterile phosphate buffer saline (PBS, pH 7.3), the prepared *Lactobacillus* and *Bacillus* powders (1 × 10^10^ cfu/g) were diluted with starch and added into the basal diet to a final concentration of 10^8^ cfu/kg, respectively. The same amount of starch was added to each group to compensate for the difference in the nutrient composition of the diets.

### Chicken Husbandry and Experimental Design

A total of 720 *Cobb* 500 broiler chicks were purchased from ZhengDa Broilers Development Center of Zhejiang University (Hangzhou, China) and reared in XinXin Broiler Farm (Jiaxing, China). These 1-day-old chicks with similar weights were randomly allocated to four groups with six replicates per group and 30 chickens per replicate. The two probiotic treatment groups received the diets consisting of Lac16 and BSC10 (10^8^ cfu·kg^−1^ feed) for 21 days, respectively, while the control group and Cp group were only fed the basal diet (**Table 1**) during the whole trial. On day 1 and days 14 to 20, birds of all groups except the control group were orally challenged with 1 × 10^8^ cfu *C. perfringens* type A strain once a day. The chickens were exposed to a 24 h lighting device and kept with ambient temperature gradually decreasing from 32°C to 26°C at the rate of 2°C per week. Broilers were provided freshwater and feed *ad libitum*. The study was carried out according to the guidelines of the Animal Care and Use Committee of Zhejiang University.

**Table 1:** Ingredients and nutrition composition of basal diet (%).

Item	1–21 d
Ingredients	
Corn	60.00
Soybean meal	28.50
Fish meal	2.00
Wheat middling	4.50
Dicalcium phosphate	1.30
Limestone	2.25
50% choline chloride	0.15
Salt	0.30
Vitamin and mineral premix1	1.00
Total	100.00
Calculated Nutrient Level	
CP	22.39
Total P	0.70
Ca	1.00
Lys	1.17
Met + Cys	0.65
Met	0.48
ME (MJ/kg)	12.22

^1^Premix compound each kilogram contained: vitamin A, 12,500 IU; vitamin D, 32,500 IU; vitamin E, 18.75 IU; vitamin K, 32.65 mg; vitamin B2, 6 mg; vitamin B12, 0.025 mg; Biotin, 0.0325 mg; Folic acid, 1.25 mg; Nicotinic acid, 50 mg; vitamin B3, 12 mg; Cu, 8 mg; Fe 80 mg; Zn, 75 mg; Mn, 100 mg; Se, 0.15 mg; I, 10.35 mg.

### Sample Collection and Treatment

At the end of the experimental period, two chickens per cage with a weight close to the average of the group were selected and immediately slaughtered by exsanguination. The intestine was collected by simultaneously washing with cold sterile PBS to remove the attached impurity, and the jejunal mucosa and ileum mucosa were gently scraped. Jejunal samples were diluted with nine-time volumes of sterile ice-cold normal saline (0.9%) based on the sample weight and then homogenized using a hand-held glass homogenizer. The tissue supernatants were collected by centrifuging at 3,500×g for 10 min at 4°C, and the concentration of protein was determined by a BCA protein assay kit according to the manufacturer’s instruction (Pierce, Rockford, IL) and stored at −80°C for further study.

### Ileal Morphology and Immunohistochemistry

Approximately 1 cm of distal ileum was dissected for histomorphology analysis. Ileal tissues were fixed (in 4% paraformaldehyde overnight), dehydrated, and embedded in paraffin according to the standard procedure (18). The paraffin-embedded tissues were cut into 5 µm thick and subsequently subjected to hematoxylin and eosin staining and observed by a light microscope (Nikon Eclipse80i, Tokyo, Japan). The immunohistochemistry staining was carried out as described previously with minor modification (18). Briefly, after dewaxing and rehydration, the tissue sections underwent microwave antigen retrieval in sodium citrate buffer (0.01 M, pH 6.0) for 20 min. Endogenous peroxidase activity was blocked with 3% hydrogen peroxide and then using 10% goat serum (ZSGB-BIO, Beijing, China) as a non-specific binding block for 30 min at room temperature. The sections were incubated overnight at 4°C with polyclonal rabbit antibody Bax (BIOSS, Beijing, China) or BCl-2 (BIOSS, Beijing, China). The tissues were incubated with Biotinylated secondary antibodies (Polink-2 plus polymer HRP anti-rabbit or anti-mouse, ZSGB-BIO, Beijing, China) and further visualized with a diaminobenzidine-tetrachloride (DAB) kit (TIANGEN RA110, Beijing, China). All sections were counterstained with hematoxylin for 3 min.

### TUNEL Assay

Apoptosis in ileum tissue was analyzed by the terminal dUTP-nick end labeling kit (Roche, Germany). Briefly, the paraffin-embedded tissue sections of ileum were incubated with proteinase K working solution at 37°C for 25 min and washed three times with PBS, and then incubated with permeabilization solution for 20 min. Following three times with PBS, the samples were incubated with TUNEL reaction mixture for 60 min at 37°C in a humidified chamber and strained with DAPI for 10 min and rinsed with PBS for three times. The cell nuclei were identified using UV light microscopy (Nikon, Japan) and TUNEL-positive cells were identified as brilliant green.

### RNA Extraction and RT-qPCR

The RNA was extracted using Takara RNAiso Plus Kit (Japan) following the manufacturer’s protocol. The purity and concentration of total RNA were determined using a Nanodrop Spectrophotometer (ND-2000, Thermo Fisher Scientific). The reverse transcription of total RNA was performed by Reverse Transcriptase M-MLV Kit (RNase H-) according to the manufacturer’s instruction. The qRT-PCR assay was conducted with the ABI 7500 fluorescence detection system using SYBR green (Takara, SYBR Premix Ex Taq TM II Kit) detection. Each sample was measured in duplicate. Relative quantitation of all gene expression was calculated using the 2^-△△Ct^ method, and the *β*-actin served as the internal reference gene (19). Primers used in the current study were designed using the Primer Express 3.0 software (Applied Biosystems, Foster City, CA), and the specificity of primers was assessed by melting curve analysis. Primers are listed in **Table 2**.

**Table 2:** Primers used in the experiment.

Gene	Primer sequence (5′–3′)	Accession number
*Bax*	GTGATGGCATGGGACATAGCTC	XM_015274882.1
	TGGCGTAGACCTTGCGGATAA	
*P53*	CCCATCCTCACCATCCTTACA	XM_420232
	CTTCAGCATCTCATAGCGGC	
*Occludin*	TCATCCTGCTCTGCCTCATCT	NM_205128.1
	CATCCGCCACGTTCTTCAC	
*Claudin1*	CATACTCCTGGGTCTGGTTGGT	NM_001013611.2
	GACAGCCATCCGCATCTTCT	
*MUC-2*	TTCATGATGCCTGCTCTTGTG	XM_421035
	CCTGAGCCTTGGTACATTCTTGT	
*ZO-1*	CTTCAGGTGTTTCTCTTCCTCCTC	XM_413773
	CTGTGGTTTCATGGCTGGATC	
*Caspase-9*	TCAGACATCGTATCCTCCA	XM_424580.6
	AAGTCACAGCAGGGACA	
*Caspase-3*	ACTCTGGAAATTCTGCCTGATGAC	NM_204725.1
	CATCTGCATCCGTGCCTGA	
*BCL-2*	GATGACCGAGTACCTGAACC	NM_205339.2
	CAGGAGAAATCGAACAAAGGC	
*IL-1β*	CGACATCAACCAGAAGTGCTT	NM_204524.1
	GTCCAGGCGGTAGAAGATGA	
*IL-6*	CAGGACGAGATGTGCAAGAA	NM_204628.1
	TAGCACAGAGACTCGACGTT	
*IL-10*	ACCAGTCATCAGCAGAGCAT	NM_001004414
	CCTCCTCATCAGCAGGTACTC	
*TGF-β*	TCTCGGAGCAGCGGATAGA	JQ423909.1
	AATCCAAGGTTCCTGTCTCTGT	
*iNOS*	CACGTGTTAAGGATGCCCCT	NM_204961
	GCCCAATAGCCACCTTCAGT	
*IFN-γ*	ACACTGACAAGTCAAAGCCGC	NM_205149
	AGTCGTTCATCGGGAGCTTG	
*β-*actin	CAACACAGTGCTGTCTGGTGGTA	NM 205518
	ATCGTACTCCTGCTTGCTGATCC	

### Detection of iNOS Activity and NO Production

The activity of inducible nitric oxide synthase (iNOS) and production of Nitric oxide (NO) in the jejunal mucosa were measured strictly according to the manufacturer’s protocols (Jiancheng Bioengineering Institute, Nanjing, China).

### DNA Extraction and Library Construction of Cecum Content

The cecal contents of twelve chickens from four groups (Control, Cp, Cp+Lac16, Cp+BSC10) were collected on day 21. The fecal DNA was extracted using TIANamp DNA Stool Mini Kit (TianGen, Beijing), and the quality was checked by agarose gel electrophoresis. All of the extracted DNA samples were stored at −80°C for further processing.

DNA library was constructed by TruSeq Nano DNA LT Library Preparation Kit (FC-121-4001). DNA was fragmented using dsDNA Fragmentase (NEB, M0348S) by incubating at 37°C for 30 min. Library construction begins with fragmented cDNA. Blunt-end DNA fragments are generated using a combination of fill-in reactions and exonuclease activity, and size selection is performed with provided sample purification beads. An A-base is then added to the blunt ends of each strand, preparing them for ligation to the indexed adapters. Each adapter contains a T-base overhang for ligating the adapter to the A-tailed fragmented DNA. These adapters contain the full complement of sequencing primer hybridization sites for single, paired-end, and indexed reads. Single- or dual-index adapters are ligated to the fragments and the ligated products are amplified with PCR by the following conditions. The initial denaturation was at 95°C for 3 min, 8 cycles of denaturation at 98°C for 15 sec, annealing at 60°C for 15 s, and extension at 72°C for 30 s, and then final extension at 72°C for 5 min.

### Metagenomics Analysis of Taxonomic Profiling and Functional Profiling

Raw sequencing reads were processed to obtain valid reads for further analysis. Assessment of sequence quality was used in fastqc. The host genome sequence was removed using the Best Match Tagger (BMTagger), referred to the genome of gga_ref_Gallus_gallus-5.0. Using fastq_to_fasta, the fq format data was converted to fasta format, which further used the filter_fasta.py command in QIIME to remove the host genome sequence. The remaining sequence was regarded as the intestinal flora DNA sequence for further analysis. The species composition was analyzed using MetaPhlAn2 (20), the cladogram was conducted with GraPhlAn, and the heatmap was drawn with metaphlan_hclust_heatmap.py The functional profiling of microbiota was analyzed using HUMAnN2, referred to the uniref90_ec_filtered_diamond database and the uniref50_ec_filtered_diamond database (21).

Principal component analysis of gene family was performed using prompt R package. Heatmap of metabolic pathway abundance was drawn using the fold-change value (log2 transformed) using the pheatmap R package. The Kyoto Encyclopedia of Genes and Genomes (KEGG) pathway was analyzed using the KEGG online service tools’ KEGG mapper.

### Statistical Analysis

All data were analyzed by a one-way analysis of variance (ANOVA) followed by Tukey multiple comparisons procedure using SPSS 22.0 software (SPSS Inc., Chicago, IL, USA). Statistical significance was declared at *P* < 0.05 and trend at *P* < 0.1. The data were expressed as mean ± SD, and graphs were generated by GraphPad Prism 7.0 software. Statistical analyses and data visualization for fecal microbiota were conducted using the R program (version 3.6.1).

## Results

### Lac16 and BSC10 Alleviated Intestinal Mucosal Injury Induced by *C. perfringens* Infection

**Figure 1:**
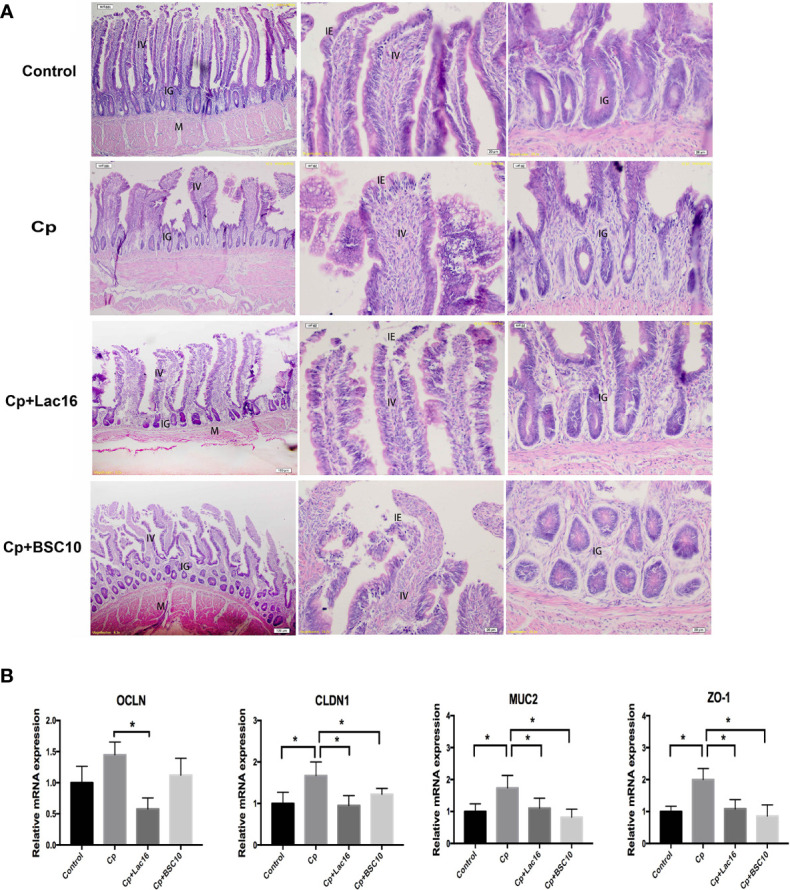
Probiotics *Lactobacillus plantarum* 16 (Lac16) and *Paenibacillus polymyxa* 10 (BSC10) alleviated intestinal mucosal injury induced by *Clostridium perfringens* (Cp) infection. **(A)** From the left to the right column, hematoxylin–eosin (HE) staining showing the integral morphology of the ileum, the villus, and the grand in the Control, Cp, Cp *+* Lac16, and Cp + BSC10 groups. Magnification, ×100, ×200, and ×200, respectively. IV, intestinal villus; IE, intestinal epithelium; IG, intestinal glands; M, muscularis. n = 6/group. **(B)** The mRNA expression of barrier-related genes assessed by RT-PCR. CLDN, Claudin; OCLN1, Occludin 1; ZO-1, Zonula occludins-1; MUC2, Mucin 2. Data were expressed as mean ± SD (n = 12). *indicates statistically significant difference (*P* < 0.05).

Hematoxylin–eosin staining showed that the damaged intestinal structure and shorter villus were observed in *C. perfringens*-infected birds, which were recovered by probiotics treatment (**Figure 1A**). Besides, the *C. perfringens* infection significantly increased the expression of intestinal barrier-related genes, such as *claudin1* (*CLDN1*), *occludin*-1 (*ZO-1*), and *mucin*-2 (*MUC2*) in the ileum (*P <* 0.05). However, the expression of *OCLN* in the Cp + Lac16 group was significantly lower compared with the Cp group (**Figure 1B**).

### Lac16 and BSC10 Attenuated Apoptosis Induced by *C. perfringens* Infection

**Figure 2:**
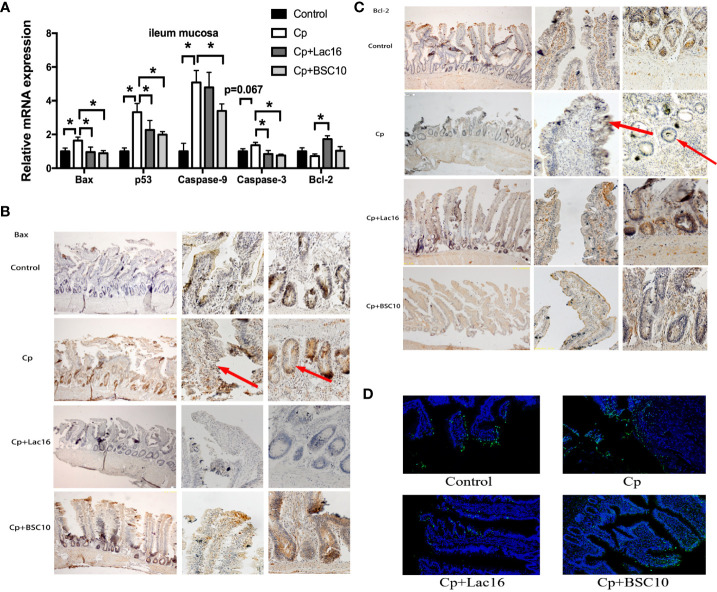
Probiotics treatment attenuated apoptosis induced by *Clostridium perfringens* (Cp) infection. The apoptosis-related gene expression in the ileum mucosa **(A)** (n = 12/group). Representative images of the immunohistochemical staining of Bax **(B)** and Bcl-2 **(C)** in the ileum mucosa (n = 6/group). The positive cells are stained brown. From the left to the right column, the integral morphology of the ileum, the villus, and the grand are seen (magnification ×100, × 400, and × 400, respectively). **(D)** TUNEL immunofluorescence data showing the distribution of apoptosis cells (brilliant green) in the ileum mucosa. Magnification, × 200. n = 6/group. * indicates statistically significant difference ( P < 0.05).

The mRNA expression levels of pro-apoptosis genes in the ileum mucosa, including *Bax, p53*, *Caspase-9* were up-regulated in *C. perfringens*-infected birds (*P <* 0.05). However, Lac16 and BSC10 treatment downregulated the expression levels of the pro-apoptosis genes except for *Caspase-9* in the Lac16 group (*P <* 0.05). Moreover, a significant increase in *Bcl-2* mRNA level was observed in the Lac16 group compared to the *C. perfringens*-infected birds (*P <* 0.05) (**Figure 2A**). The result of Bax immunohistochemistry demonstrated that compared with the control group, *C. perfringens* infection increased the number of Bax positive cells, while Lac16 restored them to a normal level. BSC10 also showed a slight decrease in the number of Bax positive cells compared with the Cp group (**Figure 2B**). *C. perfringens* infection significantly decreased the number of Bcl-2-positive cells, while probiotic treatment alleviated the decrease (**Figure 2C**). TUNEL assay results demonstrated that *C. perfringens* infection enhanced the number of positive cells in the ileum, while there was a decline following Lac16 or BSC10 treatment (**Figure 2D**).

### Lac16 and BSC10 Down-Regulated *C. perfringens-*Induced Inflammatory Response

**Figure 3:**
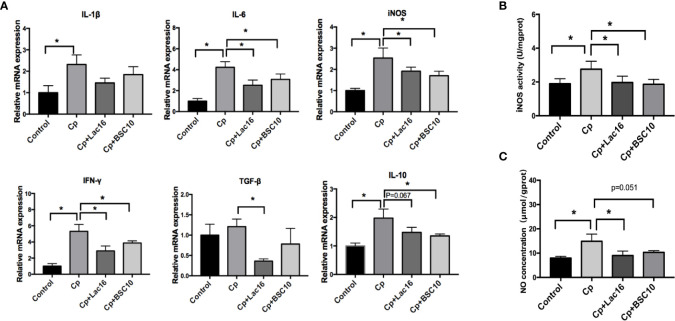
*Lactobacillus plantarum* 16 (Lac16) and *Paenibacillus polymyxa* 10 (BSC10) down-regulated the *C. perfringens-*induced inflammatory response. **(A)** cytokines gene expression profiles in the ileum mucosa determined by RT-qPCR. The iNOS activity **(B)** and NO production **(C)** in jejunal mucosa were measured using commercial kits. Results are presented as mean ± SD (n = 12/group, *represents significant differences (*P* < 0.05).

*C. perfringens* infection increased the expression of anti-inflammatory cytokine *IL-10* and pro-inflammatory cytokines, *IFN-γ, IL-6, IL-1β*, and *iNOS* (*P <* 0.05) (**Figure 3A**), while Lac16 or BSC10 treatment significantly decreased the expression levels of *IL-6* (*P <* 0.05)*, iNOS* (*P <* 0.05), *IFN-γ* (*P <* 0.05), and *IL-10* (*P <* 0.1 and *P <* 0.05, respectively). Compared with the Cp group, Lac16 supplementation down-regulated *TGF-β* expression (*P <* 0.05). Nitric oxide (NO) and inducible nitric oxide synthase (iNOS) actively participated in the host defense in response to *C. perfringens* infection. As depicted in **Figures 3B, C**, the highest iNOS activity and NO production in the jejunal mucosa was observed in the Cp group but decreased following Lac16 or BSC10 treatment.

### Lac16 and BSC10 Re-Shifted the Intestinal Microbiota Composition in Cp-Infected Broilers

**Figure 4:**
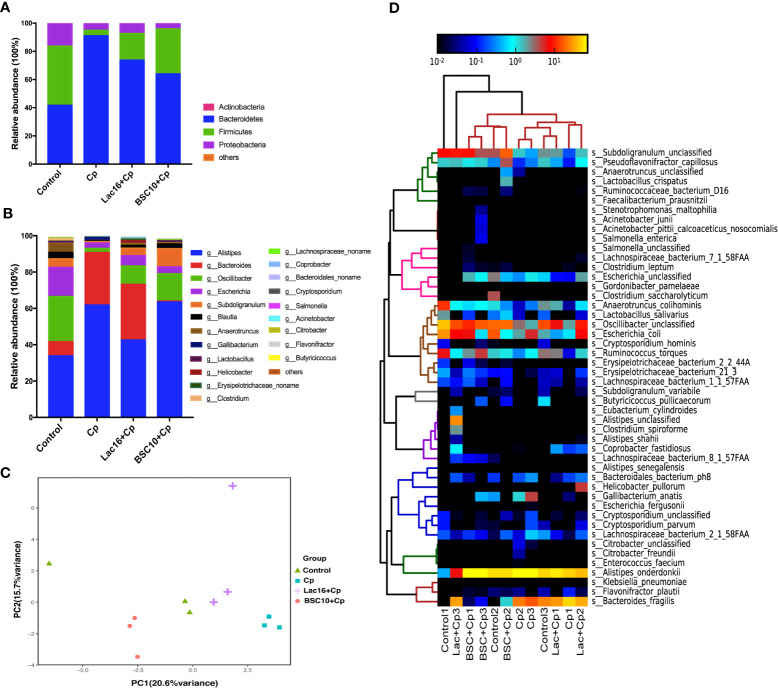
Bacterial taxonomic composition of cecum contents. **(A)** The relative abundance of bacterial phyla (mean of each group); **(B)** The top 15 genus abundance (mean of each group); **(C)** Principal component analysis of the dissimilarity among the microbial samples using prompt R package; **(D)** Heatmap of species abundance by MetaPhlAn2. n = 3/group.

As shown in **Supplemental Figure S1**, phylogenetic analysis revealed the most abundant composition of fecal microbiota at the family, genus, and species levels among all groups. At the family level, *Rikenellaceae, Bacteroidaceae*, and *Oscillospiraceae* were dominant. The composition of gut microbiota at the phylum level is shown in **Figure 4A**. The predominant phyla were *Firmicutes, Bacteroidetes*, and *Proteobacteria*, which were more than 99%. *C. perfringens* infection decreased the relative abundance of *Firmicutes* and *Proteobacteria*, and increased *Bacteroidetes*, while probiotic treatment increased the relative abundance of *Firmicutes* and decreased *Bacteroidetes*. At the genus level, the gut microbiota was dominated by genus *Allistipes*, accounting for 50.41%, followed by *Bacteroides* and *Oscillibacter*, accounting for 16.97 and 13.08%, respectively. Lac16 or BSC10 treatment enhanced the relative abundance of *Oscillibacter* but decreased *Allistipes* and *Bacteroides* (**Figure 4B**). Principal Components Analysis (PCA) showed the different clusters of microbial communities among the four groups, with PC1 accounting for 20.6% of the total variation and PC2 accounting for 15.7% (**Figure 4C**). Four bacterial species that were enriched in all groups included, *Alistipes, Bacteroides, Osciliibacter*, and *Escherichia* (**Figure 4D**). *C. perfringens* infection enriched *Gallibacterium anatis* and those that decreased included *Erysipellotrichaceac, Subdoligranulum, Anaerotruncus, Ruminococcus, Pseudoflavonifractor*, and *Oscillibacter*, which were found to be increased in the probiotic treatment groups. The abundance of *Bacteroides fragilis* was significantly decreased in the Cp + BSC10 group, compared with the Cp and Cp + Lac16 group.

### Functional Capacity of the Gut Microbiome Related to Metabolic Pathways

**Figure 5:**
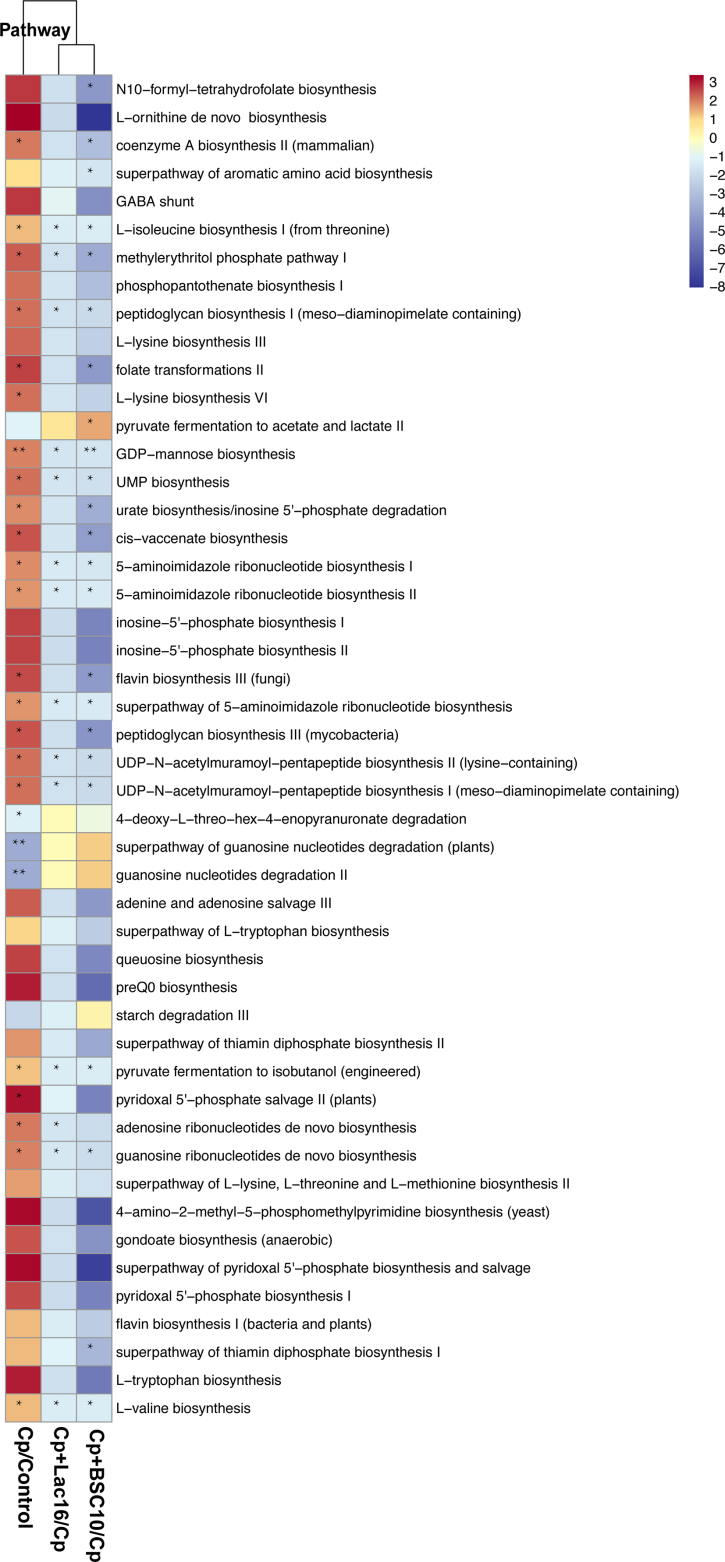
The functional profile of cecum contents. Differences in metabolic pathways expression of the microbiota among different treatment groups evaluated using the HUMAnN2 metabolic analysis network. The representative metabolic pathway is selected by a one-way ANOVA test, and the fold changes of pathway abundance were calculated using the formula Cp/Control, Cp + Lac16/Cp, and Cp + BSC10/Cp. The significant difference is defined as the fold change > 2 or fold change < 0.5. A heatmap is drawn using the fold-change value (log_2_ transformed) of the metabolic pathway abundance using the pheatmap R package. * indicates statistically significant difference (P < 0.05).

The functional capacity of the gut microbiome was evaluated using metagenomic sequencing data. We classified the predicted genes by aligning and assigning them to pathways using the HUMAnN2 database. PCA analysis of the gene family abundance revealed dramatic changes between the Cp group and other groups (**Supplemental Figure S2**). *C. perfringens* infection changed the microbiota composition and metabolic pathways, including enhancing B-vitamin biosynthesis (for example, folate biosynthesis as shown in **Supplemental Figure S3**), peptidoglycan biosynthesis (**Supplemental Figure S4**), amino acid biosynthesis and ribonucleotide biosynthesis, *etc*, and decreased pyruvate fermentation to acetate and lactate II pathway, which was reversed by probiotic supplementation (**Figure 5**).

## Discussion

Numerous studies have demonstrated that probiotics exert antimicrobial activity against *C. perfringens* through different mechanisms. In our previous study, increased intestinal lesion scores and decreased growth performance of broilers were observed after *C. perfringens* challenge, which was ameliorated in broilers pretreated with Lac16 and BSC10 (unpublished data). Therefore, the current study aimed to investigate the protective effects of Lac16 and BSC10 against *C. perfringens* in broilers through the regulation of the intestinal mucosal structure, apoptosis, inflammation, and intestinal microbiota.

Gut integrity is a prerequisite for maintaining the host homeostasis. The intestinal mucosal barrier comprises connecting epithelial cells that are overlaid by host-secreted mucous layer and serve as the first line of defense against pathogens and potentially harmful commensal bacteria (22, 23). Impaired intestinal mucosal barrier caused by intestinal pathogens compromises the immune tolerance of the intestines and causes a systemic inflammatory response, which aggravates systemic immune response and host body damage (24). Consistent with a previous study (25), the present study demonstrated that probiotics Lac16 and BSC10 treatment could improve the intestinal morphology of *C. perfringens* infected broilers. MUC2 is the main component of mucins which binds several pathogens and inhibits external bacterial access to the epithelial cells (26). Previous studies have shown that the expression of MUC2 is suppressed in infected broilers (27, 28). However, Fasina et al. (29) reported that *Salmonella Typhimurium* infection increased the number of goblet cells and mucin level in the jejunum of broilers, indicating that the infected birds can capture and eliminate the pathogens from the epithelial surface by enhancing mucin expression (30). We also found that the MUC2 expression was significantly increased in *C. perfringens* infected broilers but decreased after probiotic supplementation. Our results may unravel novel strategy for improving mucus barrier to inhibit *C. perfringens* infection. Occludin and claudin are two of the most important components of tight junctions, and they exhibit beneficial effects on epithelial barrier function (31). Similar with the result of MUC2 expression, the present study showed that *C. perfringens* infection significantly up-regulated the relative expression levels of ZO-1 and CLDN1, which was contrary to results from previous studies (32, 33). One plausible explanation was that up-regulating the expression levels of barrier-related genes in infected chicken was beneficial for decreasing *C. perfringens* to pass through the epithelial layers and intestinal damage. However, the probiotic supplementation could decrease the number of *C. perfringens* and improve intestinal health status, which resulted in the decreased expression of occludin and claudin.

Apoptosis is regulated by the dynamic balance between the expression of pro-apoptosis proteins, such as Bax, and the anti-apoptosis protein, such as Bcl-2. Once the imbalance occurs, cytochrome c is released from the mitochondria and subsequently activates Apaf1/Caspase 9 and downstream executioner Caspase 3, thereby initiating cell apoptosis (34). p53 is a multiple function protein that induces apoptosis by promoting the expression of pro-apoptosis genes, such as Bax, and inhibits anti-apoptosis genes, such as survivin, thereby activating the caspase-dependent pathway and ultimately triggering apoptosis (35). Wu et al. (36) reported that *Lactobacillus rhamnosus* GG stimulates apoptosis by increasing p53 expression and decreasing Bcl-2 and Bcl-xl protein in the ilea of HRV-infected pigs. In the present study, the results of mRNA expression and immunohistochemistry of apoptosis-related genes showed that the probiotics could ameliorate *C. perfringens*-induced apoptosis in the ileum by up-regulating anti-apoptosis genes and down-regulating pro-apoptosis genes in broilers, and these findings were consistent with previous findings (37). Moreover, p53 mRNA expression in the probiotics groups was down-regulated compared with the Cp group, suggesting that the probiotics inhibited cell apoptosis in the ileum through the p53 signaling pathway during *C. perfringens* infection.

Accumulating evidence demonstrates that iNOS-derived NO and pro-inflammatory cytokines exert multiple modulatory effects on the host immune response against various infections (38, 39). Besides, pro-inflammatory cytokines modulate host immunity against multiple pathogens through differentiation and proliferation of immune cells, apoptosis, and NO production (40). However, excessive inflammatory responses cause tissue injury. The increase in NO production and iNOS activity and increased expression of pro-inflammatory cytokines, such as *IL-1β, IL-6, iNOS*, and *IFN-γ* demonstrate that *C. perfringens* infection induces a strong inflammatory response and causes severe tissue damage (41). However, in the present study, probiotics reduced the inflammatory response, thus inhibiting excessive inflammatory damage in infected broilers. Similar results have also been observed in birds infected with *Salmonella enteritidis* (42) and *Escherichia coli* (43). Interleukin-10 is an anti-inflammatory cytokine that inhibits T cell proliferation and the production and function of many proinflammatory cytokines (44). In the present study, the level of *IL-10* decreased following treatment with the probiotics in *C. perfringens* infected broilers, and this was attributed to a decreased inflammatory response.

The microbiota plays a major role in host growth and health and significantly contributes to the regulation of the host immune system, nutrient synthesis, energy metabolism, and prevention of enteric pathogen infection (45). The gut microbial composition and function are reported to be modulated by dietary probiotic supplementation (46). The increase in *Firmicutes* contributes to energy efficiency, while the *Firmicutes/Bacteroides* ratio is associated with growth performance and host health (47, 48). A high proportion of *Proteobacteria* in the gut is an indicator of metabolic disorders, immune disorders, and an unstable gut microbial community structure in the host (49). In the present study, increased *Firmicutes/Bacteroidetes* ratio and a decline in *Proteobacteria* were associated with improved intestinal health in broilers supplemented with probiotics.

*Bacteroides fragilis* is generally regarded as a gut commensal and leads to an increased risk of infection and disease when it leaks into the bloodstream or surrounding tissue (50). Even though *Bacteroides fragilis* has an anti-inflammatory property, toxigenic *B. fragilis* induces intestinal inflammation and can cause bowel disease and colon cancer (51). *Gallibacterium* is an indigenous bacterial pathogen in chicken and one of the major pathogens causing reproductive tract disorders in laying hens (52). Butyrate is an important energy source for gut enterocytes, and can reduce the inflammatory response and gastrointestinal pathogens, regulate the gut bacterial ecology, and stimulate villi growth (53, 54). Two probiotics increased some beneficial bacteria and butyrate-producing bacteria, such as *Ruminococcus, Oscillibacter, Pseudoflavonifractor*, and *Erysipellotrichaceac and* decreased *Gallibacterium* in *C. perfringens-*infected birds. In addition, BSC10 decreased *Bacteroides fragilis*. Together with the results of the PCA, we speculated that the two probiotics could restore the intestinal microbiota disturbance induced by *C. perfringens* infection with minor differences.

Alterations in the intestinal microflora composition are closely related to the metabolic alterations in the gut microbiota. In the present study, *C. perfringens* infection changed the microbiota composition and metabolic pathways, including enhancing B-vitamin biosynthesis, peptidoglycan biosynthesis, amino acid biosynthesis, and ribonucleotide biosynthesis, etc., and decreased pyruvate fermentation to acetate and lactate II pathway, which were reversed by probiotic supplementation. Acetate and lactate are known to inhibit the growth of gut pathogens, suggesting that probiotics exert antibacterial properties by increasing the production of acetate and lactate in response to *C. perfringens*. B-vitamins are important in numerous metabolism processes, including fat and carbohydrate metabolism and DNA synthesis. Hosts cannot produce enough B-vitamins and have to be supplemented either from the diet or the gut microbiota (55). An increased risk of colitis correlates with a deficiency of genetic pathways involved in polyamine transport and B vitamin biosynthesis (56, 57). Dubin et al. (58) found that three modules involved in the biosynthesis of B vitamins (riboflavin (B2), pantothenate (B5), and thiamine (B1)) were more abundant in colitis free patients. Besides, Ford et al. (59) reported that high-dose vitamin-B supplementation reduced oxidative stress and inflammation. Inconsistent with some previous studies, the present study showed that the vitamin-B biosynthetic pathway was enriched in the Cp group but decreased in the probiotics group. We speculated that *C. perfringens* induced a compensatory synthesis of vitamin-B against oxidative damage and inflammation, while *Lactobacillus* and *Bacillus* synthesized vitamin-B in the gut to maintain normal levels in the organism, hence no need to supply exogenous Vitamin-B after probiotic treatment. Peptidoglycan (PGN) is a component of the cell wall in both Gram-positive and Gram-negative bacteria, and it triggers inflammatory responses through multiple pattern-recognition receptors (60). The most well-defined sensors of the peptidoglycan are NOD-like receptors (NLRs), which promote pathogen clearance by inducing the secretion of pro-inflammatory cytokines and chemokines and other host defense pathways, including autophagy (61). Peptidoglycan predominantly induces the Th1 immune response. Besides, bacteria-derived peptidoglycan can activate Paneth cells to produce defensins that protect the host from pathogenic bacteria (62). Therefore, in the present study, downregulation of the peptidoglycan biosynthetic pathway in the probiotic groups indicated that probiotics might protect against *C. perfringens*-infections by inhibiting excessive inflammatory response, and these findings were also consistent with the results of cytokines expression levels in the ileum mucosa.

## Conclusion

In our study, the two probiotics have similar molecular mechanism against *C. perfringens* infection, including improving the composition and metabolic pathways of the intestinal microbiota, intestinal structure, inflammation, and anti-apoptosis. There are some differences in anti-apoptosis and the composition and metabolic pathways of the intestinal microbiota of these two probiotics.

## Data Availability Statement

The datasets presented in this study can be found in online repositories. The names of the repository/repositories and accession number(s) can be found below: https://www.ncbi.nlm.nih.gov/sra/PRJNA675467.

## Ethics Statement

The animal study was reviewed and approved by the Animal Care and Use Committee of Zhejiang University.

## Author Contributions

WL, LG, and HZ conceived and designed the experiments. LG and BW performed the experiments. LG and WL analyzed the data. LG, LT, and ZZ made the figures. LG and WL wrote the paper. WL, YZ, and HZ revised the manuscript. All authors contributed to the article and approved the submitted version.

## Funding

This study is supported by the National Natural Science Foundation of China (No. 31472128 and 31672460), the Natural Science Foundation of Zhejiang Province (No. LZ20C170002), National High-Tech R&D Program (863) of China (No. 2013AA102803D), the Major Science and Technology Project of Zhejiang Province (No. 2006C12086), PRC.

## Conflict of Interest

The authors declare that the research was conducted in the absence of any commercial or financial relationships that could be construed as a potential conflict of interest.

## Supplementary Material

Supplementary Figure 1 Phylogenetic analysis of the microbiota composition.

Supplementary Figure 2 Principal component analysis of the gene family using the prompt R package.

Supplementary Figure 3 KEGG map of the folate biosynthesis (map 00790) pathway. The fold changes of pathway abundance are calculated using the formula Cp/Control, Cp + Lac16/Cp, and Cp + BSC10/Cp. The significant difference is defined as the fold change >2 or fold change <0.5. The up-regulated, fold changes are labeled as green, blue, and purple, respectively, while the down-regulated fold changes are labeled as red, yellow, and pink, respectively. The brown color represents the collection of green, yellow, and pink, while the gray color represents the collection of red, blue, and purple.

Supplementary Figure 4 KEGG map of the peptidoglycan biosynthetic (map 00550) pathway. The fold changes of pathway abundance are calculated using the formula Cp/Control, Cp + Lac16/Cp, and Cp + BSC10/Cp. The significant difference is defined as the fold change >2 or folds change <0.5. The up-regulated fold changes are labeled as green, blue, and purple, while the downregulated are labeled as red, yellow, and pink. The brown color represents the collection of green, yellow, and pink, while the gray color represents the collection of red, blue, and purple.
