# Supplementary figures and images for: Protective Effects of Lactobacillus plantarum 16 and Paenibacillus polymyxa 10 Against Clostridium perfringens Infection in Broilers
Source: Front Immunol. 2021 Feb 18;11:628374. doi: 10.3389/fimmu.2020.628374 (PMC7930238; doi:10.3389/fimmu.2020.628374)

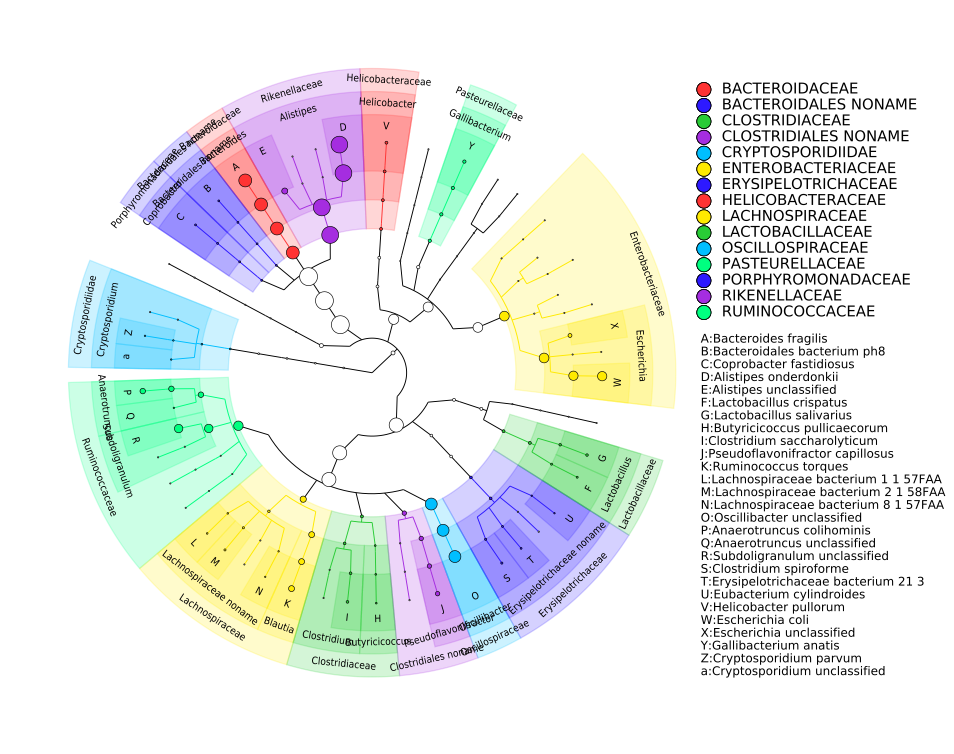

Supplement: Supplementary Figure 1 — Phylogenetic analysis of the microbiota composition. [file Image_1.png]

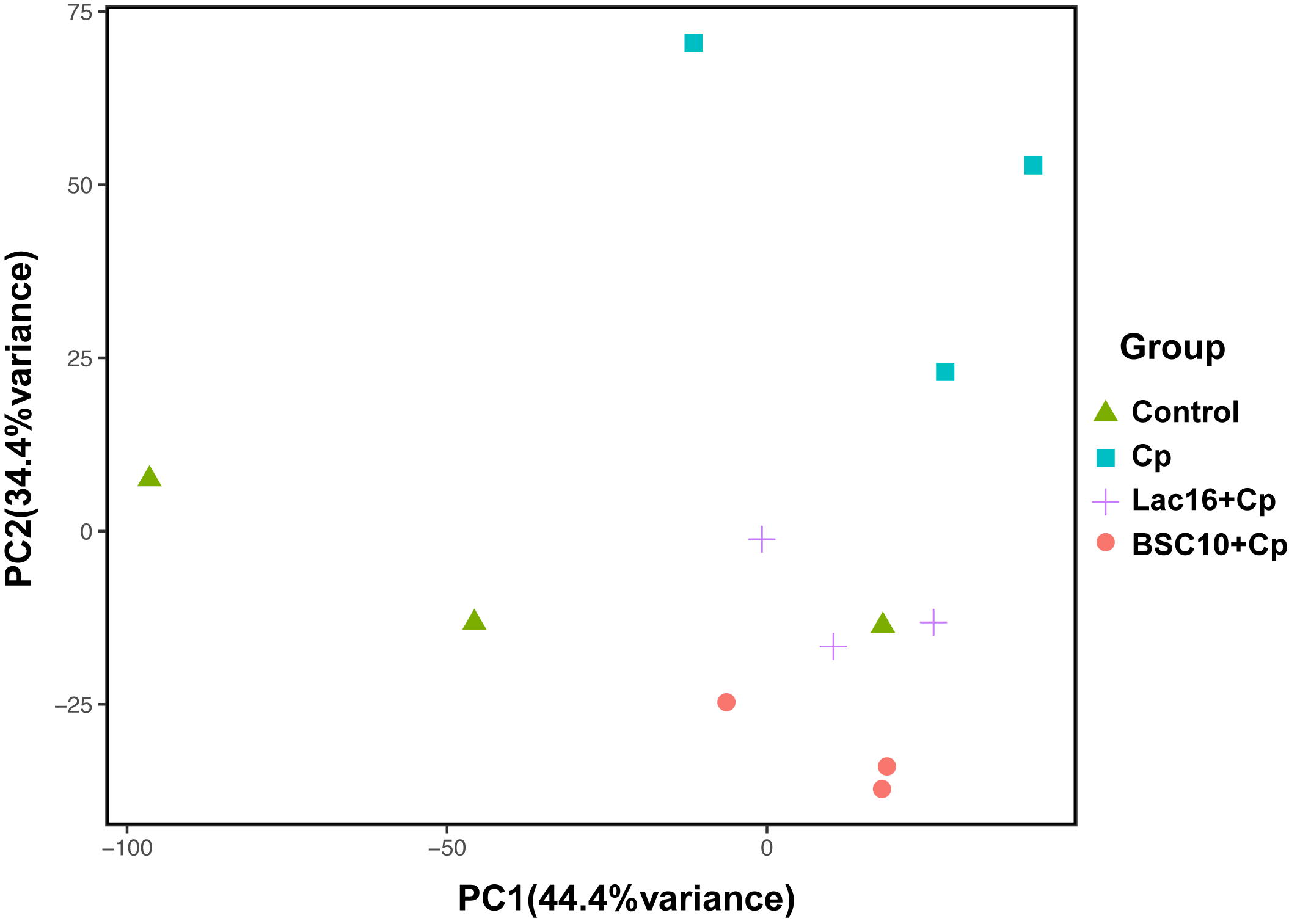

Supplement: Supplementary Figure 2 — Principal component analysis of the gene family using the prompt R package. [file Image_2.tif]

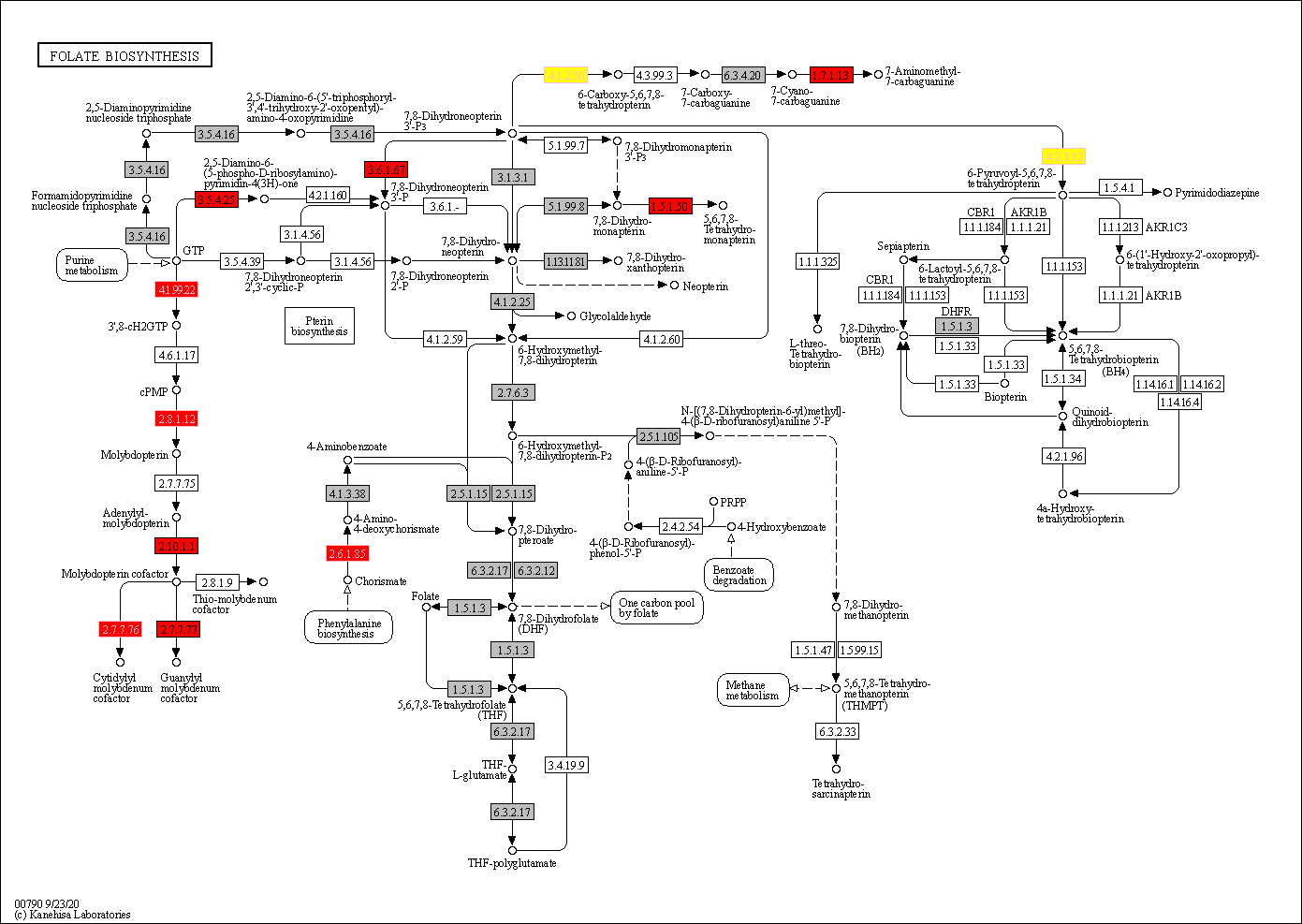

Supplement: Supplementary Figure 3 — KEGG map of the folate biosynthesis (map 00790) pathway. The fold changes of pathway abundance are calculated using the formula Cp/Control, Cp + Lac16/Cp, and Cp + BSC10/Cp. The significant difference is defined as the fold change >2 or fold change <0.5. The up-regulated, fold changes are labeled as green, blue, and purple, respectively, while the down-regulated fold changes are labeled as red, yellow, and pink, respectively. The brown color represents the collection of green, yellow, and pink, while the gray color represents the collection of red, blue, and purple. [file Image_3.png]

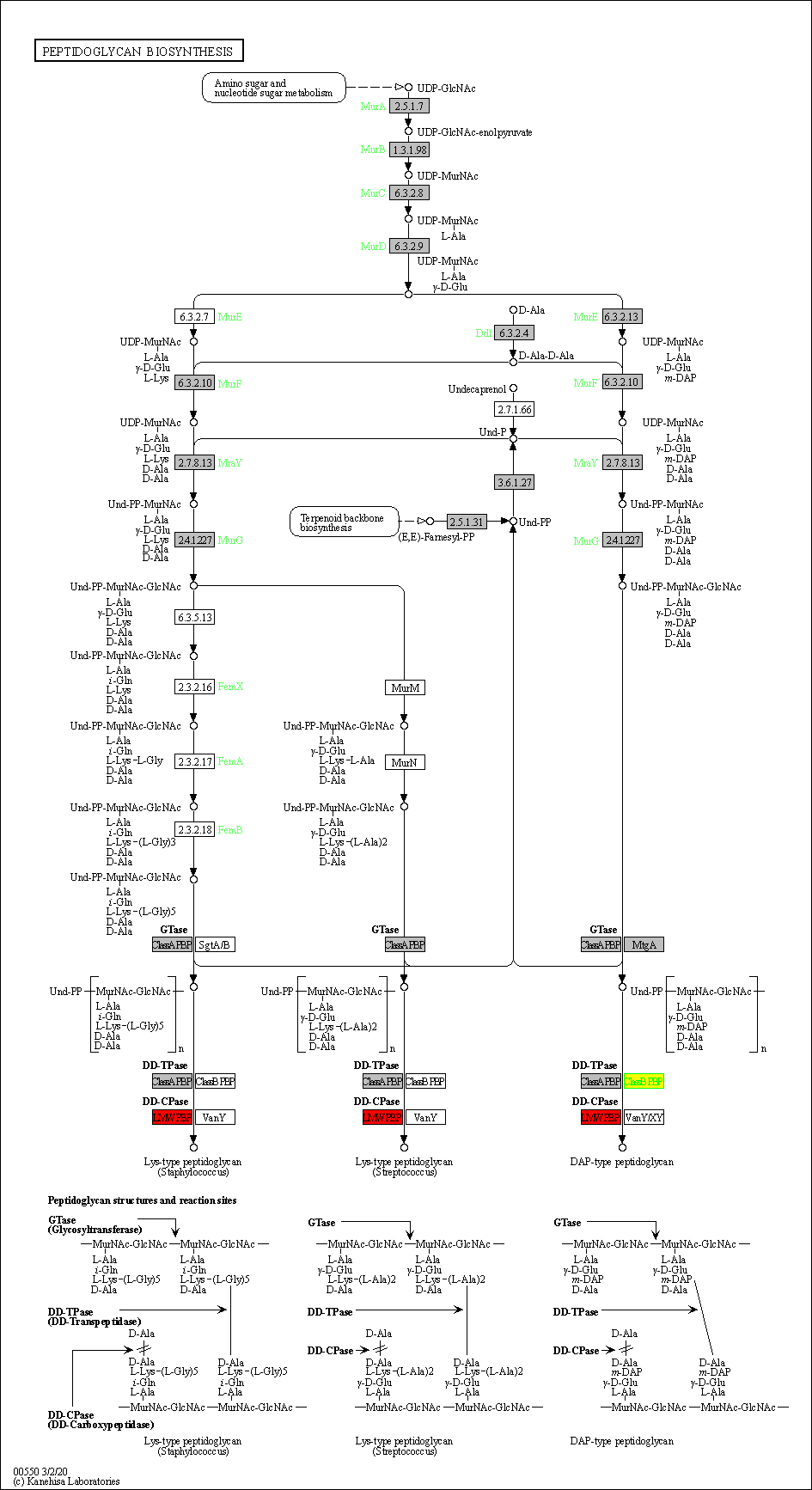

Supplement: Supplementary Figure 4 — KEGG map of the peptidoglycan biosynthetic (map 00550) pathway. The fold changes of pathway abundance are calculated using the formula Cp/Control, Cp + Lac16/Cp, and Cp + BSC10/Cp. The significant difference is defined as the fold change >2 or folds change <0.5. The up-regulated fold changes are labeled as green, blue, and purple, while the downregulated are labeled as red, yellow, and pink. The brown color represents the collection of green, yellow, and pink, while the gray color represents the collection of red, blue, and purple. [file Image_4.png]
